# Cigarette smoke induces endoplasmic reticulum stress and suppresses efferocytosis through the activation of RhoA

**DOI:** 10.1038/s41598-020-69610-x

**Published:** 2020-07-28

**Authors:** Hiroyuki Ito, Yoshiro Yamashita, Takeshi Tanaka, Masahiro Takaki, Minh Nhat Le, Lay-Myint Yoshida, Konosuke Morimoto

**Affiliations:** 1 Department of Clinical Medicine, Institute of Tropical Medicine, Nagasaki University, 1-12-4 Sakamoto, Nagasaki City, Nagasaki 852-8523 Japan; 2 Department of Clinical Tropical Medicine, Nagasaki University Graduate School of Biomedical Sciences, Nagasaki, Japan; 3 Department of Pediatric Infectious Diseases, Institute of Tropical Medicine, Nagasaki University, Nagasaki, Japan

**Keywords:** Apoptosis, Endoplasmic reticulum

## Abstract

Impaired efferocytosis is a key mechanism of inflammatory lung diseases, including chronic obstructive pulmonary disease and cystic fibrosis. Cigarette smoking activates RhoA and impairs efferocytosis in alveolar macrophages, but the mechanism has not been fully elucidated. We investigated the role of endoplasmic reticulum (ER) stress induced by cigarette smoking in the disruption of efferocytosis. Both tunicamycin (10 μg/ml) and thapsigargin (0.1 and 1 μM), which are ER stress inducers, suppressed efferocytosis in J774 cells, and a Rho-associated coiled-coil-forming kinase (ROCK) inhibitor (Y27632) reversed this effect. We validated the effect of tunicamycin on efferocytosis in experiments using RAW264.7 cells. Then, we investigated the role of the unfolded protein response (UPR) in efferocytosis impaired by ER stress. A PERK inhibitor (GSK2606414) restored the efferocytosis that had been impaired by TM, and an eIF2α dephosphorylation inhibitor (salubrinal) suppressed efferocytosis. Cigarette smoke extract (CSE) induced ER stress in J774 macrophages and RhoA activation in J774 cells, and the CSE-induced ROCK activity was successfully reversed by GSK2606414 and tauroursodeoxycholic acid. Finally, we confirmed that ER stress suppresses efferocytosis in murine alveolar macrophages and that GSK2606414 could rescue this process. These data suggest that cigarette smoke-induced ER stress and the UPR play crucial roles in RhoA activation and suppression of efferocytosis in the lung.

## Introduction

Efferocytosis is a fundamental process by which apoptotic cells are recognized and removed by professional and nonprofessional phagocytes. Apoptotic cell removal is critical for homeostasis because it suppresses inflammation and promotes tissue repair via the production of growth factors, such as hepatocyte growth factor (HGF)^1^. Efferocytosis has been found to be impaired in multiple chronic respiratory diseases (e.g., chronic obstructive pulmonary disease (COPD), cystic fibrosis and asthma), and it is thought to contribute to their pathogenesis by enhancing inflammation and impairing tissue repair^2–6^. The regulation of efferocytosis is tightly controlled by small Rho-GTPases, such as RhoA and Rac1^7–9^. We previously reported that cigarette smoking (CS)-associated oxidative stress activates RhoA and impairs efferocytosis in murine alveolar macrophages (AMs)^10^, but the mechanism remains to be elucidated.

Endoplasmic reticulum (ER) stress is induced in cells that exhibit excessive synthesis and abnormal accumulation of proteins in the ER lumen^11^. Under such conditions, the ER initiates the unfolded protein response (UPR) to resolve the protein folding defect and protect the cell. If the protein folding defect persists, ER stress initiates programmed cell death, i.e., apoptosis. ER stress has been shown to contribute to the pathogenesis of systemic diseases, such as diabetes and atherosclerosis^12,13^, and chronic lung diseases, such as pulmonary fibrosis and COPD^14–18^. CS has been shown to induce ER stress in epithelial cells and cause apoptosis, which is also observed in patients with COPD^17,19^. Cash et al*.* reported that ER stress decreases efferocytosis via peritoneal macrophages in an apolipoprotein E4 mouse model^20^^,^ however, the mechanism was not investigated. Although AMs are directly exposed to CS, the effect of CS-induced ER stress on efferocytosis by AMs has not been investigated. Therefore, in this study, we examined whether CS-induced ER stress impairs efferocytosis by activating RhoA.

## Results

### ER stress impaired efferocytosis in the macrophage cell lines J774 and RAW264.7

**Figure 1:**
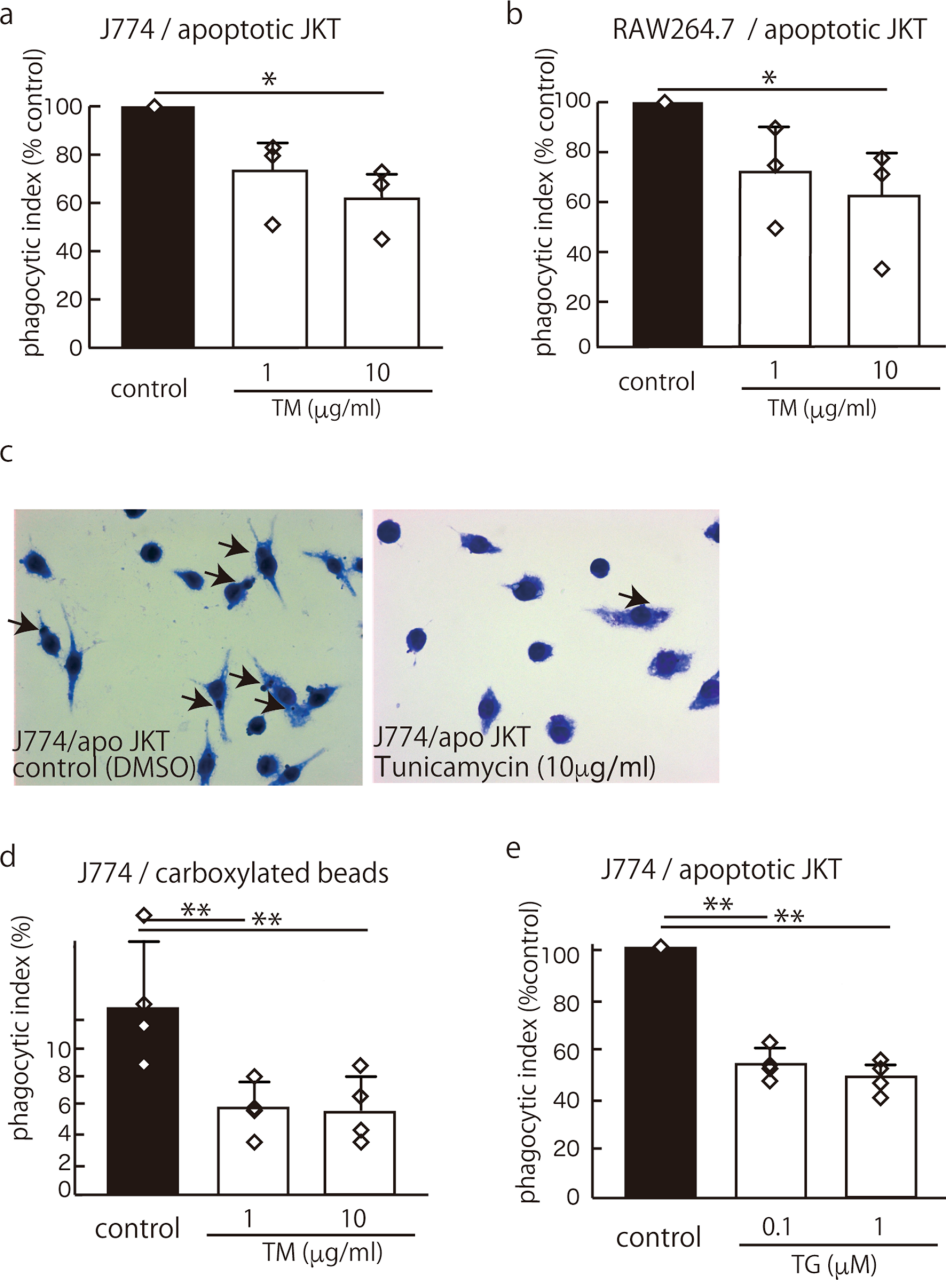
ER stress caused impaired efferocytosis. After J774 cells (**a**, **c**) or RAW264.7 cells (**b**) were stimulated with 10 μg/ml TM for 6 h, UV-induced apoptotic Jurkat cells or carboxylated beads (**d**) were added. The mean PI is shown as a percentage of the control ± SEM of three to four replicates per group. The statistical analysis was performed using an ANOVA, followed by Dunnett’s test to compare the groups with an internal control when the ANOVA indicated significance. (**a**) TM significantly suppressed efferocytosis in the J774 cells (*p < 0.05) (control mean PI, 12.9 ± 3.3) (n = 3). (**b**) TM similarly suppressed efferocytosis in the RAW264.7 cells (*p < 0.05) (control mean PI, 3.6 ± 2.7) (n = 3). (**c**) Representative photomicrographs of Diff Quik-stained J774 cells (magnification, ×100) with ingested apoptotic Jurkat cells (arrows). (**d**) TM (10 μg/ml for 6 h) also significantly inhibited the phagocytosis of carboxylated beads by J774 cells (**p < 0.01) (control mean PI, 12.9 ± 4.4) (n = 4). (**e**) TG, which induces ER stress by inhibiting an endoplasmic reticulum Ca^2+^ ATPase inhibitor, also suppressed efferocytosis in the J774 cells (**p < 0.01) (control mean PI, 19.4 ± 14.8) (n = 4).

As previously reported, 6 h of treatment with tunicamycin (TM), an antibiotic known to promote ER stress by blocking N-linked protein glycosylation, induced the expression of the UPR genes BiP, CHOP and sXBP-1 in both J774 and RAW264.7 macrophages (Fig. S1a, b). Then, we tested the effect of ER stress on efferocytosis. UV-induced apoptotic Jurkat cells were added to J774 cells and RAW264.7 cells that were treated with 10 μg/ml TM for 6 h. The results showed that 10 μg/ml TM significantly suppressed efferocytosis in the J774 cells and the RAW267.7 cells (Fig. 1a, b, c). We also tested the effect of TM (treatment with 10 μg/ml for 6 h) on the phagocytosis of carboxylated beads in J774 cells and found that TM significantly suppressed their phagocytosis (Fig. 1d). Thapsigargin (TG), which induces ER stress by inhibiting an endoplasmic reticulum Ca^2+^ ATPase inhibitor, also suppressed efferocytosis in the J774 cells (Fig. 1e). TM had no effect on the viability of the J774 cells and the RAW264.7 cells 24 h after the treatment, as measured by the MTS Cell Proliferation Assay Kit (Fig. S2a, b). TG also had no effect on the viability of J774 cells subjected to the same assay (Fig. S2c).

### ER stress suppressed efferocytosis in a RhoA/ROCK-dependent manner

**Figure 2:**
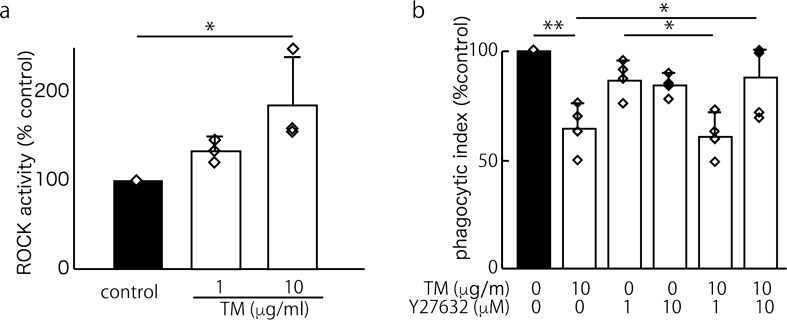
ER stress caused impaired efferocytosis in J774 cells in a ROCK/RhoA activation-dependent manner. (**a**) The induction of RhoA/Rho-kinase by TM (an antibiotic that promotes ER stress by blocking N-linked protein glycosylation) in J774 cells was evaluated using a ROCK activity assay kit. Six hours of stimulation with 1 or 10 μg/ml TM caused ROCK activation in a dose-dependent manner. The means were analyzed using an ANOVA; when the ANOVA indicated significance, Dunnett’s test was used to compare the groups with an internal control (n = 3) (*p < 0.05). (**b**) Y27632 (10 μM; a ROCK inhibitor) completely reversed the 10 μg/ml TM-induced impairment of efferocytosis (control mean PI, 19.7 ± 11.6). The mean PI is shown as a percentage of the control ± SEM of four replicates per group. The means were analyzed using an ANOVA, and when the ANOVA indicated significance, Tukey’s test was used to compare two conditions (*p < 0.05, **p < 0.01).

RhoA activation is known to suppress efferocytosis in macrophages^10^. Subsequently, we sought to determine whether TM increases the RhoA/ROCK pathway. To address this question, we exposed J774 macrophages to TM and measured the RhoA activity. We found that the treatment with 1 or 10 μg/ml TM increased RhoA activation in a dose-dependent manner (Fig. 2a). To confirm that TM suppresses efferocytosis in a RhoA/ROCK-dependent manner, we tested whether Y27632 can rescue J774 cells from impaired efferocytosis in the presence of TM (10 μg/ml). As shown in Fig. 2b, 10 μM Y27632 completely reversed the TM-induced efferocytosis impairment.

### The PERK-eIF2α pathway plays a crucial role in impairing efferocytosis through ER stress

**Figure 3:**
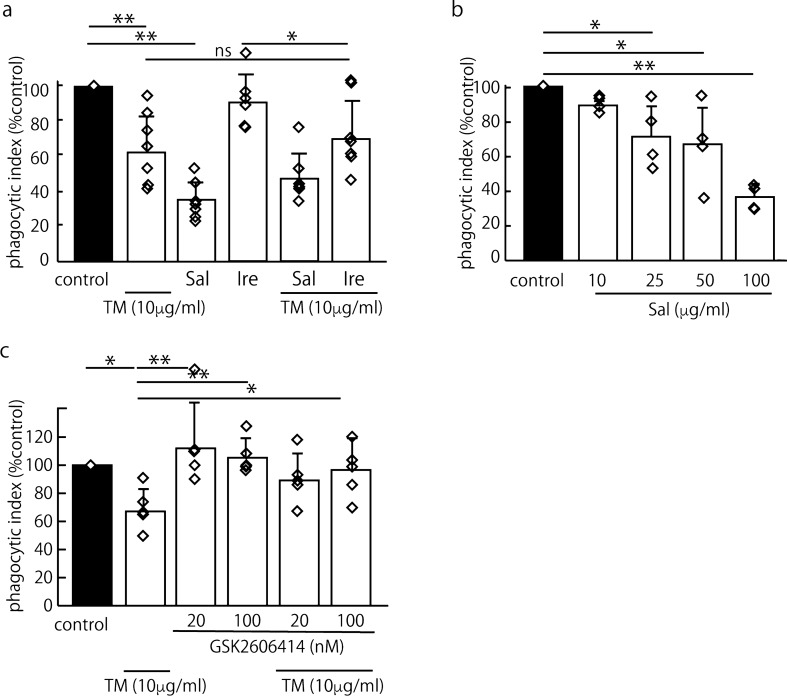
ER stress impaired efferocytosis through the PERK/eIF2α pathway. To identify the pathway contributing to RhoA activation, we tested the effects of an eIF2α dephosphorylation inhibitor (salubrinal; Sal), an IRE1 inhibitor (irestatin 9389; Ire) and a PERK inhibitor (GSK2606414) on efferocytosis in TM-treated J744 cells. The mean PI is shown as a percentage of the control ± SEM three, four or seven replicates per group. The statistical analysis was performed using an ANOVA, followed by Dunnett’s test to compare the groups with an internal control (**b**) or Tukey’s test to compare two conditions (**a**, **c**, **d**) when the ANOVA indicated significance (*p < 0.05, **p < 0.01). (**a**) Salubrinal (100 μM) strongly suppressed efferocytosis (**p < 0.01, ANOVA and Tukey’s test). Irestatin 9389 (2.5 μM) had no effect on TM-treated cells (control mean PI, 37.3 ± 7.4) (n = 7). (**b**) Salubrinal suppressed efferocytosis in naïve J774 cells in a dose-dependent manner (control mean PI, 19.5 ± 4.5) (n = 4). (**c**) To confirm the roles of PERK and eIF2α in the impairment of efferocytosis by ER stress, we tested the effect of GSK2606414, a PERK inhibitor. We found that 20 nM GSK2606414 clearly reversed the impaired efferocytosis under ER stress in a dose-dependent manner (control mean PI, 16.6 ± 1.5) (n = 5).

ER stress initiates three pathways to clear unfolded proteins and restore ER homeostasis, thus activating PERK, activating transcription factor 6 (ATF6) and inositol-requiring enzyme 1 (IRE1), which are UPR proteins^11^. First, we investigated secreted protein acidic and rich in cysteine (SPARC), which is regulated by IRE1 under ER stress, because SPARC is known to activate RhoA^21,22^. However, SPARC expression was not increased in the J774 cells after the TM stimulation as determined by western blotting (data not shown). Then, we tested the effects of an eIF2α dephosphorylation inhibitor (salubrinal, protects cells from ER stress-induced apoptosis, EC50 =  ~ 15 μM) ^23^ and an IRE1 inhibitor (irestatin 9389)^24^ on TM-treated J744 cells. Interestingly, 100 μM salubrinal strongly suppressed efferocytosis, whereas 2.5 μM irestatin 9389^24^ had no effect on efferocytosis in naïve and TM-treated cells (Fig. 3a). Then, we hypothesized that the PERK/eIF2α pathway plays a role in RhoA activation and tested the effect of salubrinal (10–100 μM) on efferocytosis in naïve J744 cells. Salubrinal suppressed efferocytosis in naïve J774 cells in a dose-dependent manner (Fig. 3b). This finding suggests that PERK/eIF2α plays a crucial role in the mechanism by which ER stress impairs efferocytosis. Subsequently, we tested the effect of the PERK inhibitor GSK2606414^25^ on TM-induced efferocytosis impairment and found that GSK2606414 clearly reversed the impairment under ER stress in a dose-dependent manner (Fig. 3c). Salubrinal and GSK2606414 had no effect on the viability of the J774 cells 24 h after the treatment, as measured by the MTS Cell Proliferation Assay Kit (Fig. S2d, e).

### GSK2606414 and TUDCA decreased cigarette smoke extract (CSE)-induced RhoA activation

To assess the cigarette smoke extract (CSE)-mediated induction of the mRNA expression of UPR signaling molecules associated with ER stress, we exposed J774 macrophages to increasing concentrations of CSE for 6 h and measured the induction of UPR signaling genes by real-time PCR. We found that CSE increased the expression of UPR genes BiP, CHOP and sXBP-1 in a dose-dependent manner and that twenty percent CSE was adequate for the experiments (Fig. S3a–c). Then, we exposed cells to twenty percent CSE for 3, 6 and 12 h (Fig. S3d–f), and both CHOP and sXBP-1 were significantly increased by the stimulation with 20% CSE for longer than 3 h.

**Figure 4:**
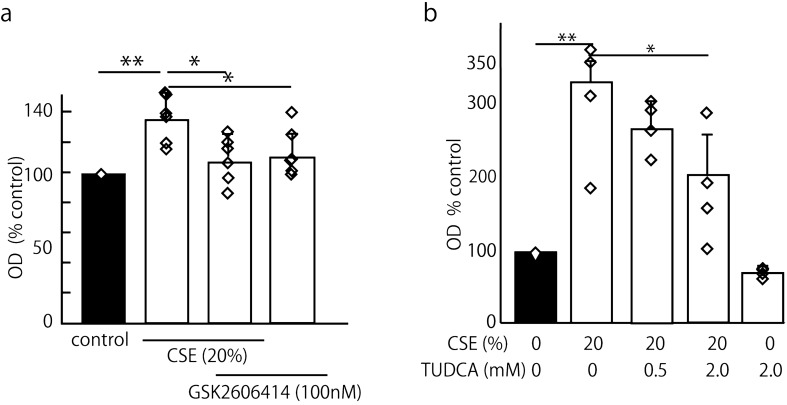
GSK2606414 and TUDCA decreased the CSE-induced RhoA activation. We evaluated whether GSK2606414 and TUDCA, which act as chemical chaperones and antioxidants, could rescue the CSE (20%)-induced RhoA activation. The mean OD is shown as a percentage of the control ± SEM of four to six replicates per group. The means were analyzed using an ANOVA, and when the ANOVA indicated significance, Tukey’s test was used to compare two conditions (*p < 0.05, **p < 0.01). (**a**) ROCK activity was measured in J774 cells pretreated with GSK2606414 for 30 min, followed by 20% CSE with or without GSK2606414 for 6 h. GSK2606414 successfully reversed the CSE-activated RhoA (135.9 ± 17.9% of the control value versus 111.9 ± 14.8% of the control value) (n = 6). (**b**) The effect of TUDCA on RhoA activation by CSE stimulation in J774 cells was tested. ROCK activity was measured in cells pretreated with TUDCA or DMSO for 60 min, followed by 20% CSE with or without TUDCA for 6 h. CSE increased RhoA activity (329.7 ± 75.9% of the control value), and 2 mM TUDCA significantly suppressed the CSE-induced RhoA activation (198.8 ± 73.4% of the control value) (n = 4).

Based on the findings above, we investigated whether the PERK/eIF2α pathway plays a role in RhoA activation in cigarette smoke exposure. ROCK activity was measured in J774 cells pretreated with GSK2606414 for 30 min, followed by 20% CSE with or without GSK2606414 for 6 h. We found that GSK2606414 successfully reversed the CSE-activated RhoA (Fig. 4a), suggesting that the PERK/eIF2α pathway is involved in the ability of CSE to increase ROCK activity. Then, we tested TUDCA, which is known as both a chemical chaperone and an antioxidant, because TUDCA can be safely administered to mammals. Increasing concentrations of TUDCA decreased the CSE-induced ROCK activity in a dose-dependent manner (Fig. 4b).

### TM suppressed efferocytosis in murine AMs in a PERK-eIF2α pathway-dependent manner

**Figure 5:**
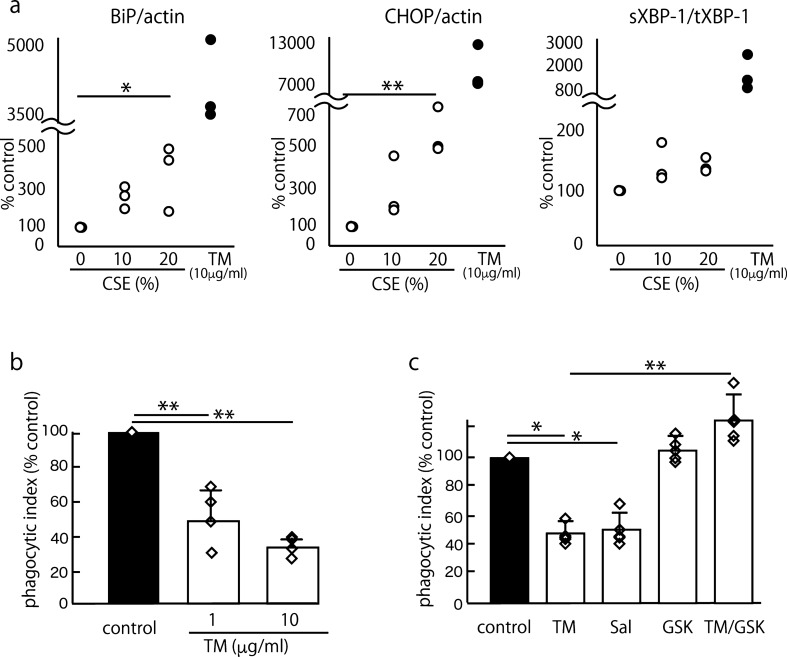
TM suppressed efferocytosis in murine AMs in a PERK-eIF2α pathway-dependent manner. To explore the impact of ER stress on efferocytosis in AMs, we performed a phagocytosis assay using AMs obtained from ICR mice and apoptotic Jurkat cells. The murine AMs were prepared by lung lavage from 8- to 10-week-old female Sic:ICR mice. The mean PI is shown as a percentage of the control ± SEM of three to four or five replicates per group. The means were analyzed using an ANOVA, and when ANOVA indicated significance, Dunnett’s test was used to compare the groups with an internal control (**a**, **b**) or Tukey’s test was used to compare two conditions (**c**) (*p < 0.05, **p < 0.01). (**a**) We confirmed the effect of TM and CSE on the induction of UPR gene expression in murine AMs. The positive control, i.e., 10 μg/ml TM, strongly induced the expression of the UPR genes BiP (4,254 ± 884% of the control value), CHOP (9,124 ± 3,289% of the control value) and sXBP-1 (1,555 ± 918% of the control value). Furthermore, 20% CSE relatively moderately increased the expression of the UPR genes BiP (374 ± 169% of the control value), CHOP (585 ± 138% of the control value), and sXBP-1 (136 ± 13.6% of the control value) (n = 3). (**b**) TM (1 and 10 μg/ml) suppressed efferocytosis in murine AMs (control mean PI, 7.85 ± 1.1%) (n = 4). (**c**) Murine AMs were treated with 100 μM salubrinal and GSK2606414 with or without 10 μg/ml TM. Consistent with our findings, salubrinal significantly suppressed efferocytosis, and GSK2606414 rescued the efferocytosis impaired by TM in murine AMs (control mean PI, 7.77 ± 1.4%) (n = 5).

To investigate whether our findings obtained using macrophage cell lines could be replicated in primary cells, we performed experiments using murine AMs collected from ICR mice. irst, we confirmed the effect of TM and CSE on the induction of UPR gene expression in murine AMs. TM (10 μg/ml) strongly induced the expression of the UPR genes BiP (4,254 ± 884% of the control value), CHOP (9,124 ± 3,289% of the control value) and sXBP-1 (1,555 ± 918% of the control value). However, CSE induced moderate changes in a dose-dependent manner, and the 20% CSE-induced mRNA expression of BiP, CHOP and sXBP-1/XBP-1 was 374 ± 169%, 585 ± 138% and 136 ± 13.6% of the control value, respectively (Fig. 5a). To explore the impact of ER stress on efferocytosis in AMs, we performed a phagocytosis assay using murine AMs and apoptotic Jurkat cells; similarly, the effect of 10 μg/ml TM was reproduced in murine AMs (Fig. 5b). TM had no effect on the viability of murine AMs 24 h after treatment, as measured by the MTS Cell Proliferation Assay Kit (Fig. S2f). Subsequently, we confirmed the roles of the PERK-eIF2α pathway in TM-impaired efferocytosis in AMs. We treated murine AMs with 10 μg/ml TM, 100 μM salubrinal and GSK2606414 with or without 10 μg/ml TM and performed an efferocytosis assay. Consistent with our findings, salubrinal significantly suppressed efferocytosis, and GSK2606414 rescued the efferocytosis impaired by TM in the murine AMs (Fig. 5c).

## Discussion

**Figure 6:**
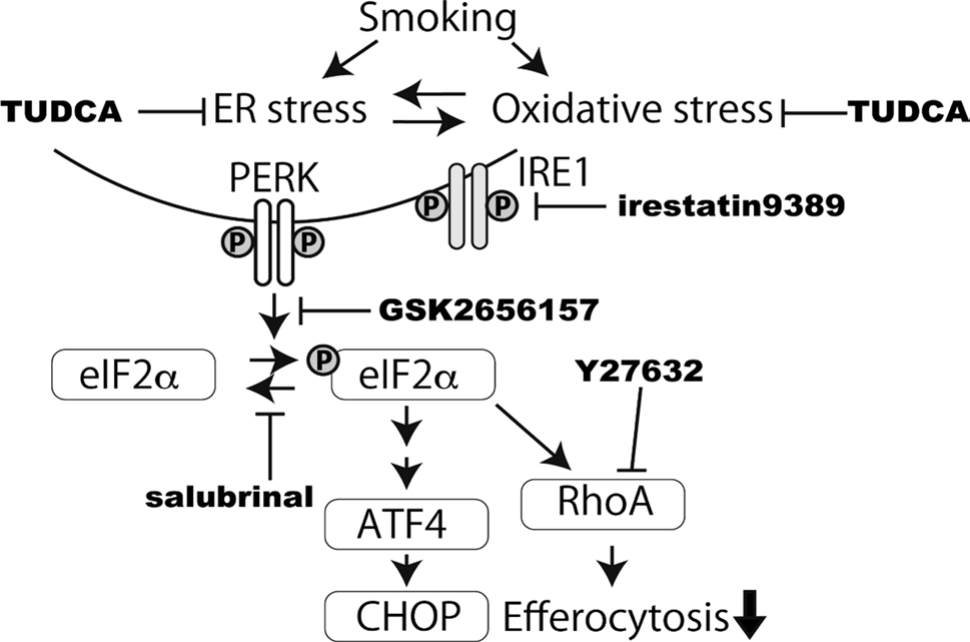
A model of the effect of the CSE-induced ER stress on efferocytosis. ER stress and oxidative stress induced by smoking activate the PERK-eIF2α pathway. The results of the experiments using TUDCA, GSK2656157 and salubrinal highlight the therapeutic potential of TUDCA in the impairment of efferocytosis by cigarette smoke.

Impaired efferocytosis is a key mechanism of inflammatory lung diseases, including COPD and idiopathic pulmonary fibrosis (IPF)^2,3,10,26,27^. Smoking is considered a risk factor for these disorders and believed to cause impaired efferocytosis by AMs via RhoA activation^10,28^. To determine whether this pathogenic process is a therapeutic target, researchers must elucidate the detailed mechanisms. In the current study, we demonstrated that ER stress activated RhoA and impaired efferocytosis through the PERK-eIF2α pathway in macrophages (Fig. 6). This finding suggests that controlling ER stress or the PERK pathway is a therapeutic strategy for these inflammatory lung diseases because PERK might be an easier molecule to target than Rho-GTPases, which play crucial roles in many cellular functions^29^.

Cigarette smoke exposure disrupts efferocytosis in macrophages in mice and human cells^10,26^, and some mechanisms have been suggested. Oxidative stress appears to impair efferocytosis by activating the RhoA/ROCK pathway^10^. Furthermore, Hodge et al. reported that antioxidative agents reversed the impairment of efferocytosis by smoke exposure^30^. ER stress and oxidative stress in chronic lung diseases are entwined with their pathogenesis^31^. Under cigarette smoke stimulation, oxidative stress causes protein misfolding through excessive protein production and oxidative protein folding interference and can eventually induce ER stress^31,32^. In the current study, we demonstrated that ER stress induced RhoA activation using TM and TG, which induce ER stress independently of oxidative stress. We also showed that CSE-induced RhoA activation was normalized by GSK2606414, a PERK inhibitor, and TUDCA. TUDCA has been known to function as not only a chemical chaperone but also an antioxidative agent in animal models of neurodegenerative disorders and ocular disorders^33–37^. Furthermore, TUDCA can be safely administered to humans and has been shown to have a pharmacological effect^38–41^. These results at least partially support the hypothesis that ER stress is a therapeutic target for cigarette smoke-related impairments in efferocytosis.

Most studies investigating the involvement of ER stress in the pathogenesis of chronic lung diseases have focused on the dysfunction or apoptosis of epithelial cells^14–18,32,42^. In the current study, we first investigated the effect of ER stress on macrophage function. We emphasized that AMs play pivotal roles in not only enhancing the inflammatory response but also controlling inflammation and tissue repair in the lungs^43^. Investigating the contribution of AMs may partially elucidate the complexity of the association between epithelial cell damage and tissue repair in cigarette smoke-affected environments. In addition to resident AMs, recruited macrophages that are differentiated from peripheral monocytes in the alveolar space during inflammation are important because they play different roles in the resolution of inflammation^44^. Since we tested the effect of ER stress in a macrophage cell line and resident murine AMs, the effects on recruited AMs in vivo remain to be elucidated.

Hodge et al. showed that the decreased expression of efferocytosis receptors under cigarette smoke stimulation results in impaired efferocytosis^26^, but we did not examine this process. The authors observed BAL macrophages obtained from patients affected by cigarette smoke and cells treated with CSE for at least 18 h in vitro. We treated the cells for only 6 h, which should be sufficient to change the expression of receptors^45^. However, we demonstrate that 10 μM Y27632, a specific inhibitor of ROCK, completely normalized the impairment in efferocytosis induced by TM. These data suggest that the impaired efferocytosis caused by cigarette smoke is associated with ER stress-induced RhoA activation. The threshold of ROCK inhibition by Y27632 to induce stress fiber formation in cultured cells seems to be between 1 and 10 μM, and the dose response curve of the effect between these two concentrations is relatively steep^46–48^. Our results show that 1 μM Y27632 had no effect, while 10 μM Y27632 completely TM-suppressed efferocytosis.

We confirmed the major findings in the current study using primary cells, i.e., murine AMs. Treatment with 1 and 10 μg/ml TM suppressed efferocytosis, and GSK2606414 reversed this effect (Fig. 5b, c). We also tested and compared the strength of 10% and 20% CSE and 10 μg/ml TM in the induction of UPR gene expression using murine AMs. While 10 μg/ml TM strongly induced UPR gene expression, CSE moderately induced these genes in a dose-dependent manner (Fig. 5a). Thus, we confirmed that the effective concentration of CSE in our experiment was 20%. We estimated that the 20% CSE used in the present study approximately corresponds to the exposure associated with smoking two packs of cigarettes per day according to a previous study^49^. However, because long-term or repetitive exposure to cigarette smoke in vivo has different effects, the investigation of in vivo models of cigarette smoking and human AMs should be the subject of future projects.

The current study has some limitations. First, we used two inhibitors, i.e., GSK2606414 and salubrinal, to investigate the roles of the PERK-eIF2α pathway in the inhibition of efferocytosis by ER stress. Recently, the PERK inhibitor GSK2606414 was revealed to inhibit TNF-a mediated RIPK1^50^ and KIT tyrosine kinase activity^51^, and salubrinal was revealed to protect antiapoptotic protein Bcl-2^52^. The nonspecific actions of these reagents might have affected our results,however, the two independent experiments yielded results supporting our hypothesis, suggesting that our conclusion is unlikely misleading. Second, the microscopic efferocytosis assay may have limitations because the J774 cells showed morphological heterogeneity (Fig. 3c). FACS has been frequently used in recent papers to evaluate efferocytosis. However, using FACS methods relying on staining apoptotic cells, it is difficult to distinguish between phagocytosis and binding. We emphasize that the activation of Rho-GTPase also affects the adhesion of cells^53^. The use of pHrodo to observe change in coloration with FACS as the pH decreases during phagocytosis is innovative^54^. pHrodo originally depends on the ingestion of nonvital latex beads. Thus, because Jurkat cells tend to form apoptotic bodies when they become apoptotic^55^, the quantification of engulfment using this probe might be challenging. A newer probe, i.e., AnnexinA5-pHrodo, shows promise as a more specific assay for the phagocytosis of apoptotic cells but is not readily available to create and use^56^.

We previously reported that 1 μg/ml TM does not significantly suppress efferocytosis in RAW264.7 cells^57^. In this previous study, we demonstrated that diabetes-induced ER stress in AMs suppresses HGF production but that efferocytosis is not significantly affected. However, in the current study, we tested higher concentrations of TM and ER stress caused by CSE, which may explain the inconsistent results. We and other groups previously showed that macrophages produce HGF during efferocytosis for tissue repair in injured lungs^1,58^. Under ER stress or CSE stimulation, various effects, including impaired efferocytosis, endoplasmic reticulum-associated degradation and inhibition of protein translation by the UPR, might cause impaired HGF production. Consistent with our previous study, we proved that HGF mRNA expression in J774 cells treated with 1 μM TM was suppressed during efferocytosis using real-time PCR (Fig. S4). Thus, cigarette smoke-induced ER stress might play crucial roles in AM dysfunction in the resolution of inflammation and tissue repair in the lungs.

## Methods

### Reagents

TM, TG and TUDCA were purchased from Sigma-Aldrich (St. Louis, MO, USA). Salubrinal and 4-phenylbutyrate were purchased from Calbiochem (San Diego, CA, USA). Y-27632, a selective ROCK inhibitor, was purchased from Wako (Osaka, Japan). GSK2606414, a protein kinase R (PKR)-like ER kinase (PERK) inhibitor (IC50 = 0.4 nM), and irestatin 9389, an IRE1 inhibitor (IC50 = 6.3 nM), were purchased from Cayman Chemical Company (Ann Arbor, MI, USA) and Axon Medchem (Groningen, the Netherlands), respectively.

### Experimental animals

Specific pathogen-free, 8- to 10-week-old female Sic:ICR mice (Charles River Laboratories Japan, Inc., Yokohama, Japan) were housed and studied under the protocols approved by Institutional Animal Care and Use Committee in Nagasaki University. The AMs were obtained from female ICR mice by lung lavage with 10 ml of ice-cold phosphate-buffered saline (PBS) containing 100 mM ethylenediaminetetraacetic acid. The harvested cells were washed twice, incubated similar to the J774 cells and used for the phagocytosis assays 24 h after harvesting.

### Cell lines, primary cells and cell culture

Murine J774.1 macrophages (J774 cells) and RAW264.7 macrophages (RAW264.7 cells) were purchased from the American Type Culture Collection (ATCC, Manassas, VA, USA) and cultured in Dulbecco’s modified Eagle’s medium (DMEM) (Gibco, Carlsbad, CA, USA) supplemented with 10% heat-inactivated fetal bovine serum (FBS), 2 mM l-glutamine, 100 mg/ml streptomycin, and 100 U/ml penicillin in humidified 5% CO_2_ at 37 °C. Human leukemia Jurkat T cells were obtained from the ATCC and cultured in RPMI 1,640 supplemented with 10% heat-inactivated FBS, 2 mM l -glutamine, 100 mg/ml streptomycin, and 100 U/ml penicillin in humidified 5% CO_2_ at 37 °C.

### Evaluation of the UPR

To confirm the presence of ER stress, we detected the mRNA levels of UPR signaling molecules by real-time RT-PCR. We measured the expression of BiP, CHOP and sXBP-1 in J774 cells and murine AMs after stimulation with CSE as described above. Real-time RT-PCR was performed as previously described. The cells (1–2 × 10^6^) were cultured in serum-free, albumin-containing DMEM in 6-well culture plates. Total RNA was extracted by using an RNeasy Mini Kit (Qiagen, Gaithersburg, MD, USA). cDNA was synthesized by using SuperScript III (Invitrogen, Carlsbad, CA, USA). The extracted mRNA was quantified by measuring the absorbance at 260 nm, and then we determined the mRNA quantity to use as a template. We mixed and incubated the reagents and templates as described in the attached protocol.

Synthesized cDNA was used as a template for real-time PCR. We used SsoFast EvaGreen Supermix with Low ROX (Bio-Rad, Hercules, CA, USA) as the reaction enzyme. The primers used for CHOP, BiP, spliced XBP-1, total XBP-1 and β-actin have been previously reported^59^. The PCR program consisted of an initial denaturation step at 50 °C for 30 s, followed by 40 cycles of denaturation at 95 °C for 5 s and annealing at 53 °C for 30 s. The PCR amplification was performed using a Light Cycler 480 (Roche, Basel, Switzerland).

### Apoptosis induction

Apoptosis was induced in Jurkat T cells by exposure to UV irradiation at 312 nm (Fotodyne, WI, USA) for 10 min as previously described^60^. After the UV irradiation, the Jurkat T cells were cultured in RPMI 1,640 without FCS for 3 h under 5% CO_2_ at 37 °C.

### Phagocytosis assay

The phagocytosis assays were performed as previously described^28,60^. Briefly, J774 cells or RAW264.7 cells were plated on glass coverslips in 24-well plates at a concentration of 3 × 10^5^ cells/well (Matsunami Glass, Osaka, Japan). When testing the effects of TM, TG and salubrinal on efferocytosis, TM or TG was added 6 h before the assay. To determine whether Y27632 can reverse the effects of TM or TG on efferocytosis, we added Y27632 to the cells 15 min before the assay. To test the effect of salubrinal on efferocytosis, we treated J774 cells for 6 h. To test whether GSK2606414 and irestatin 9389 could rescue the efferocytosis suppressed by TM, we added these compounds to the cells at the start of the stimulation with TM. Then, 3 × 10^6^ apoptotic Jurkat cells were added to each well, incubated for 60 min under 5% CO_2_ at 37 °C, and gently washed with ice-cold PBS three times to remove the undigested apoptotic cells. Then, the cells were stained with Diff Quik (Sysmex, Kobe, Japan). Phagocytosis was determined by visually inspecting the samples by oil immersion light microscopy and is expressed as the phagocytic index (PI), as previously described. Each condition was tested in duplicate, and a minimum of 200 cells per well were counted. The murine AMs were plated on 8-well Lab-Tek chamber glass slides at a concentration of 1 × 10^5^ cells/well (Thermo Fisher Scientific, Oslo Norway), and 5 × 10^5^ apoptotic Jurkat cells were used. Treatment with TM, salubrinal and GSK2606414 and the PIs of the murine AMs were evaluated similarly. In all experiments, the researcher was blinded to the sample identification during the analysis.

### Evaluation of RhoA/Rho-kinase (ROCK) activity

To evaluate the relative RhoA/Rho-Kinase (ROCK) activation in the J774 cells, we used a commercially available ROCK activity EIA kit (Cell Biolabs, Inc., San Diego, CA, USA) according to the manufacturer’s instructions. Briefly, the samples were incubated on 96-well myosin phosphatase target subunit 1 (MYPT1)-coated plates with dithiothreitol and ATP-containing kinase buffer at 30 °C for one hour. After the plate was washed, anti-phospho-MYPT1 (Thr^969^) was added and incubated. After washing, the plate was incubated with an HRP-conjugated secondary antibody and developed with a substrate solution. The absorbance was read on a spectrophotometer at a primary wavelength of 450 nm.

To prepare the samples for the ROCK activity assay, we cultured 1.5 × 10^6^ J774 cells on 6-well plates and serum-starved the cells with DMEM containing 0.2% BSA overnight. The J774 cells were treated with TM, CSE and GSK2606414 as described above, and cell lysis buffer (Cell Signaling Technology, Danvers, MA, USA) with a protease cocktail was added. The cell lysates were centrifuged at 10,000 rpm at 4 °C for five minutes, and the supernatants were stored in Eppendorf tubes (Eppendorf, Hamburg, Germany) at − 80 °C until use in the assay.

### CSE preparation

CSE was prepared as previously described^49,61^. The mainstream smoke of a commercial cigarette (Marlboro, Philip Morris, USA) was drawn by the application of a vacuum to a 50-ml plastic tube containing 20 ml of DMEM. Each cigarette was smoked for 3 min. Then, the DMEM was filtered through a 0.22-μm membrane, and the pH was adjusted to 7.4. CSE was diluted with DMEM to the designated concentrations, which were calculated with the following equation: (volume of CSE solution/total volume) × 100. According to a previous study, 20%, which is the maximum CSE concentration used in the present study, approximately corresponds to the exposure associated with smoking 2 packs of cigarettes per day^49,61^. Fresh CSE was prepared for each experiment.

### Statistics

The means were analyzed using an ANOVA for multiple comparisons; when the ANOVA indicated significance, Dunnett’s test was used to compare the groups with an internal control or Tukey’s test was used to compare two conditions. All data were analyzed using STAT (version 15) for Macintosh (Stata Corp, Texas, USA) and are presented as the mean ± SD.

## Supplementary information

Supplementary information
