# Author Correction: Cigarette smoke induces endoplasmic reticulum stress and suppresses efferocytosis through the activation of RhoA

**DOI:** 10.1038/s41598-021-85556-0

**Published:** 2021-03-08

**Authors:** Hiroyuki Ito, Yoshiro Yamashita, Takeshi Tanaka, Masahiro Takaki, Minh Nhat Le, Lay-Myint Yoshida, Konosuke Morimoto

**Affiliations:** 1 Department of Clinical Medicine, Institute of Tropical Medicine, Nagasaki University, 1-12-4 Sakamoto, Nagasaki City, Nagasaki, 852-8523 Japan; 2 Department of Clinical Tropical Medicine, Nagasaki University Graduate School of Biomedical Sciences, Nagasaki, Japan; 3 Department of Pediatric Infectious Diseases, Institute of Tropical Medicine, Nagasaki University, Nagasaki, Japan

Correction to: *Scientific Reports* 10.1038/s41598-020-69610-x, puplished online 28 July 2020

This Article contains an error in Figure 6 where the PERK inhibitor ‘GSK2606414’ is incorrectly referred to as ‘GSK2656157’. As a result, the Figure legend,

“A model of the effect of the CSE-induced ER stress on efferocytosis. ER stress and oxidative stress induced by smoking activate the PERK-eIF2α pathway. The results of the experiments using TUDCA, GSK2656157 and salubrinal highlight the therapeutic potential of TUDCA in the impairment of efferocytosis by cigarette smoke.”

should read:

“A model of the effect of the CSE-induced ER stress on efferocytosis. ER stress and oxidative stress induced by smoking activate the PERK-eIF2α pathway. The results of the experiments using TUDCA, GSK2606414 and salubrinal highlight the therapeutic potential of TUDCA in the impairment of efferocytosis by cigarette smoke.”

The correct Figure 6 appears below as Figure 1.

**Figure 1:**
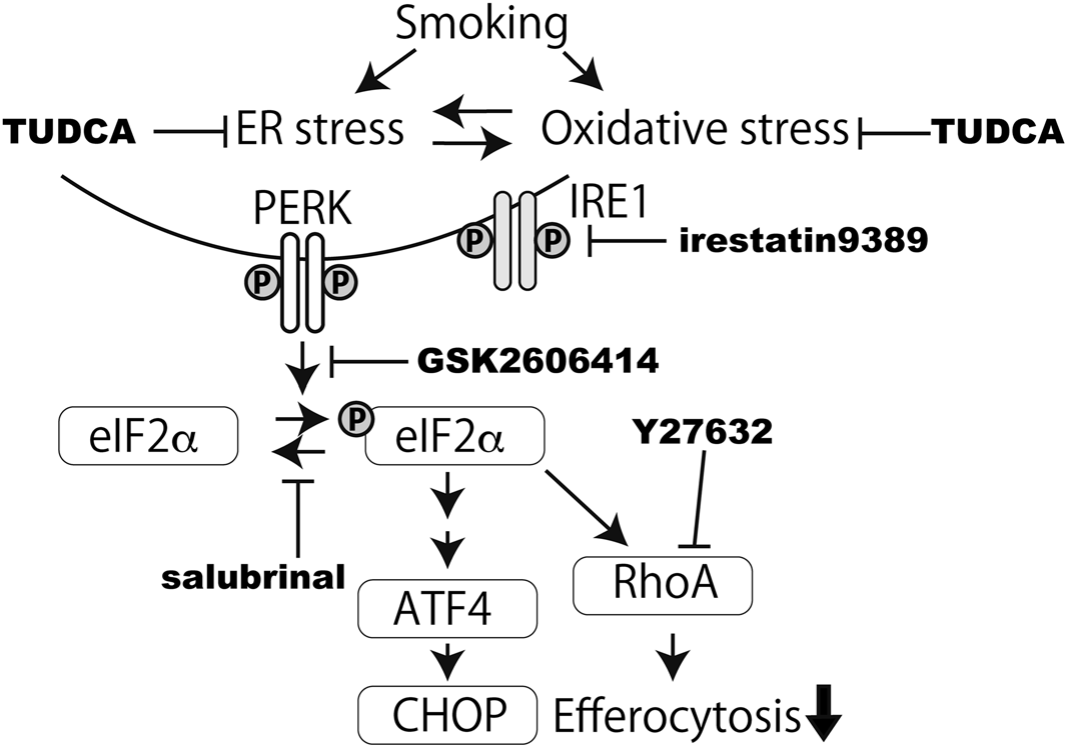
A model of the effect of the CSE-induced ER stress on efferocytosis. ER stress and oxidative stress induced by smoking activate the PERK-eIF2α pathway. The results of the experiments using TUDCA, GSK2606414 and salubrinal highlight the therapeutic potential of TUDCA in the impairment of efferocytosis by cigarette smoke.

