# Supplementary material for: Cigarette smoke induces endoplasmic reticulum stress and suppresses efferocytosis through the activation of RhoA
Source: Sci Rep. 2020 Jul 28;10:12620. doi: 10.1038/s41598-020-69610-x (PMC7387437; doi:10.1038/s41598-020-69610-x)

Cigarette smoke induces endoplasmic reticulum stress and suppresses efferocytosis through the activation of RhoA

Hiroyuki Ito^1, 2^, Yoshiro Yamashita^1^, Takeshi Tanaka^1^, Masahiro Takaki^1^, Minh Nhat Le^2^, Lay Myint Yoshida^2^ and Konosuke Morimoto^1, 2^

Author affiliations

1 Department of Clinical Medicine, Institute of Tropical Medicine, Nagasaki University, Nagasaki, Japan.

2 Department of Clinical Tropical Medicine, Nagasaki University Graduate School of Biomedical Sciences, Nagasaki, Japan.

3 Department of Pediatric Infectious Diseases, Institute of Tropical Medicine, Nagasaki University, Nagasaki, Japan.

**Supplementary methods**

## *Evaluation of the unfolded protein response (UPR)*

To confirm the presence of ER stress, we detected the mRNA levels of UPR signaling molecules by real-time RT-PCR as described in the main text (METHODS section: Evaluation of the unfolded protein response). We measured the expression of BiP, CHOP and sXBP-1 in J774 cells and RAW264.7 cells after the stimulation with 1 or 10 μg/ml TM for the designated duration. We also measured the mRNA levels of the UPR genes in murine AMs after the stimulation with 10 μg/ml TM, 10% CSE, and 20% CSE for six hours.

*Cell viability assay*

Because cytotoxic effects might affect the phagocytosis assays, we tested the viability of the cells after coculture with the indicated reagents. For the cell viability assay, we used the MTS Cell Proliferation Assay Kit (Abcam, Cambridge, UK) based on MTS tetrazolium compound reduction by viable cells to generate a colored formazan dye. According to the manufacturer’s instructions, we added 20 μl of MTS reagent to cells in 96-well plates 24 hours after coculture with the indicated reagents. Then, we measured the optical density at OD490 nm 2 hours after the start of the reaction.

*Preparation of murine AMs for the evaluation of ER stress induced by TM and CSE*

Specific pathogen-free, 8- to 10-wk-old female Sic:ICR mice (Charles River Laboratories Japan, Inc., Yokohama, Japan) were housed and studied under institutional animal care and use committee-approved protocols in the animal facility of Nagasaki University. The AMs were obtained from female ICR mice by lung lavage with 10 ml of ice-cold phosphate-buffered saline (PBS) containing 100 mM ethylenediaminetetraacetic acid. The harvested AMs were cultured with DMEM supplemented with 10% heat-inactivated fetal bovine serum (FBS), 2 mM L-glutamine, 100 mg/ml streptomycin, and 100 U/ml penicillin in humidified 5% CO_2_ at 37°C at 1×10^6^ cells/well for the real-time RT-PCR analysis of the UPR signaling components at 48 hours.

*Evaluation of hepatocyte growth factor (HGF) mRNA expression by real-time PCR*

J774 cells (2×10^6^) were treated with TM (1 μg/ml) or the control for 6 hours. After adding apoptotic Jurkat cells to the J774 cells (J774:Jurkat = 1:5), the culture was continued in DMEM supplemented with 10% FBS under 5% CO_2_ at 37°C for 14 hours with or without TM. Then, total RNA was extracted. The methods used for the RNA extraction, cDNA synthesis, and real-time PCR are the same as those described in the main text (METHODS section: Evaluation of the unfolded protein response). The mouse HGF sense and antisense primers 5'-GGACAAGATTGTTATCGTGG-3' and 5'-GTTGATCAATCCAGTGTAGC-3' were used. The primers used for β-actin have been previously reported (21). The data were evaluated by the ΔΔCq method.

**Supplementary figure legends**

Figure S1

*Tunicamycin induced ER stress in J774 cells and RAW264.7 cells*

The treatment with 10 μg/ml TM for 6 hours induced ER stress in the J774 cells (a) and RAW264.7 cells (b) as measured by a real-time PCR analysis of UPR genes. The treatment with 10 μg/ml TM for 6 hours significantly increased BiP, CHOP and spliced XBP-1 expression compared to the control. The statistical analysis was performed using an ANOVA, followed by Dunnett’s test to compare the groups with an internal control (*p<0.05, **p<0.01).

Figure S2

*Cell viability after the treatment with the indicated reagents*

The cell viability was assessed using the MST Cell Proliferation Assay Kit. The cell viability was not significantly decreased by TM, TG, salubrinal or GSK2606414 in the J774 cells, by TM in the RAW264.7 cells, or by TM in the murine AMs. The following reagent concentrations were used: a) 10 μg/ml TM in the J774 cells, b) 10 μg/ml TM in the RAW264.7 cells, c) 1 μM TG in the J774 cells, d) 100 μg/ml salubrinal in the J774 cells, e) 100 nM GSK2606414 in the J774 cells, and f) 10 μg/ml TM in the murine AMs.

Figure S3

*Cigarette smoke extract (CSE) induced ER stress in J774 cells*

The CSE-induced ER stress in the J774 cells was measured by a real-time PCR analysis of UPR genes. Six hours of CSE exposure induced the mRNA expression of UPR signaling molecules in the J774 cells in a dose-dependent manner (Fig. S3a-c). The linear regression analysis was consistent with the dose proportionality. The CSE stimulation at 20% for six hours increased the BiP/actin mRNA levels and significantly increased the CHOP/actin and spliced XBP-1/total XBP-1 mRNA levels (*p<0.05). Then, we exposed the cells to twenty percent CSE for 3, 6 and 12 hours (Fig. S3d-f), and both CHOP and sXBP-1 were significantly increased by the stimulation with 20% CSE for longer than 3 hours (*p<0.05, **p<0.01). The statistical analysis was performed using an ANOVA; when the ANOVA indicated significance, Dunnett’s test was used to compare the groups with an internal control.

Figure S4

*HGF mRNA expression following efferocytosis under ER stress*

Because TM should affect HGF glycosylation, we confirmed that HGF production was inhibited under ER stress at the mRNA level by real-time PCR. TM (1 μg/ml) significantly inhibited HGF mRNA expression following efferocytosis. The statistical analysis was performed using a Mann–Whitney *U* test (*p<0.05).


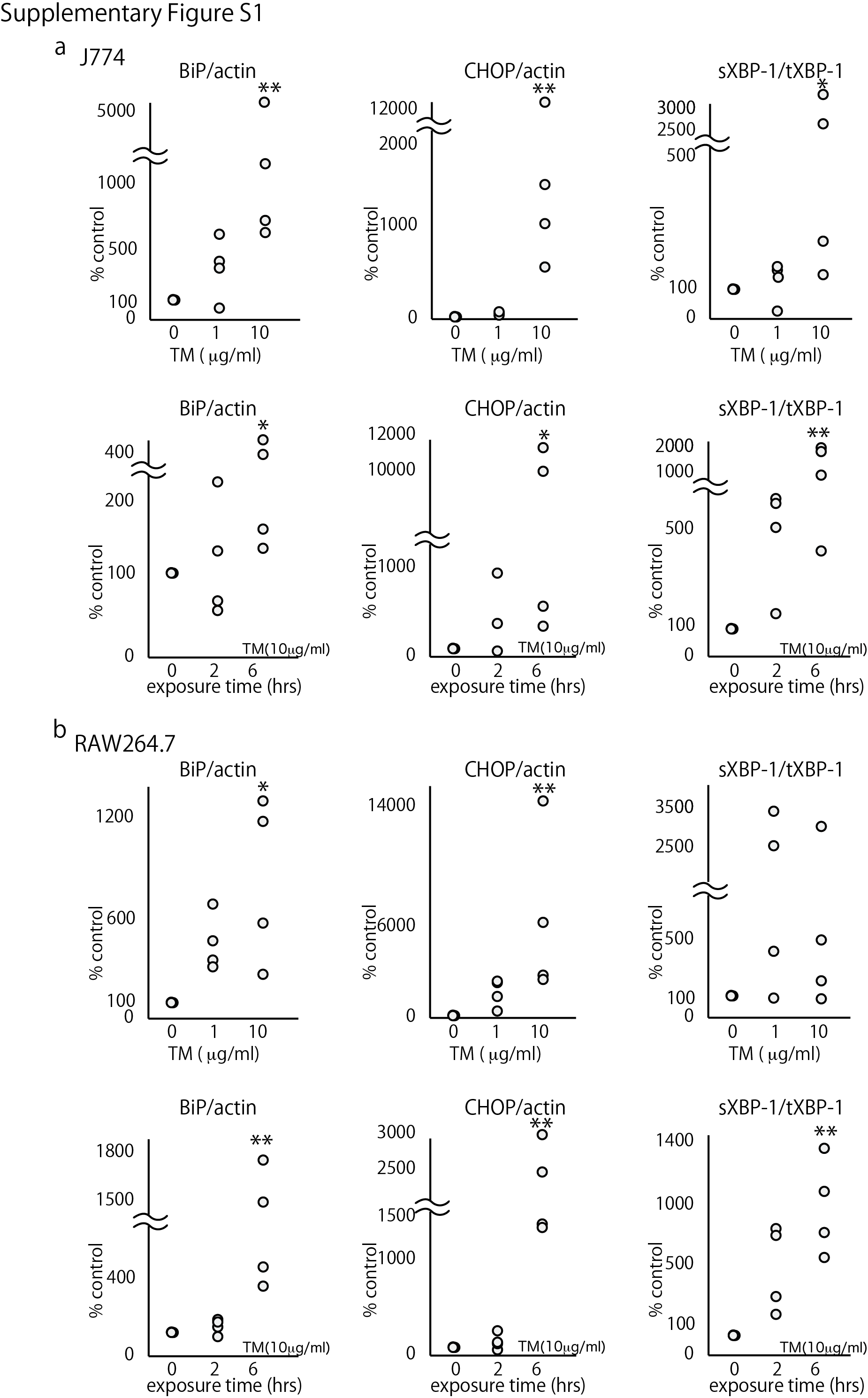


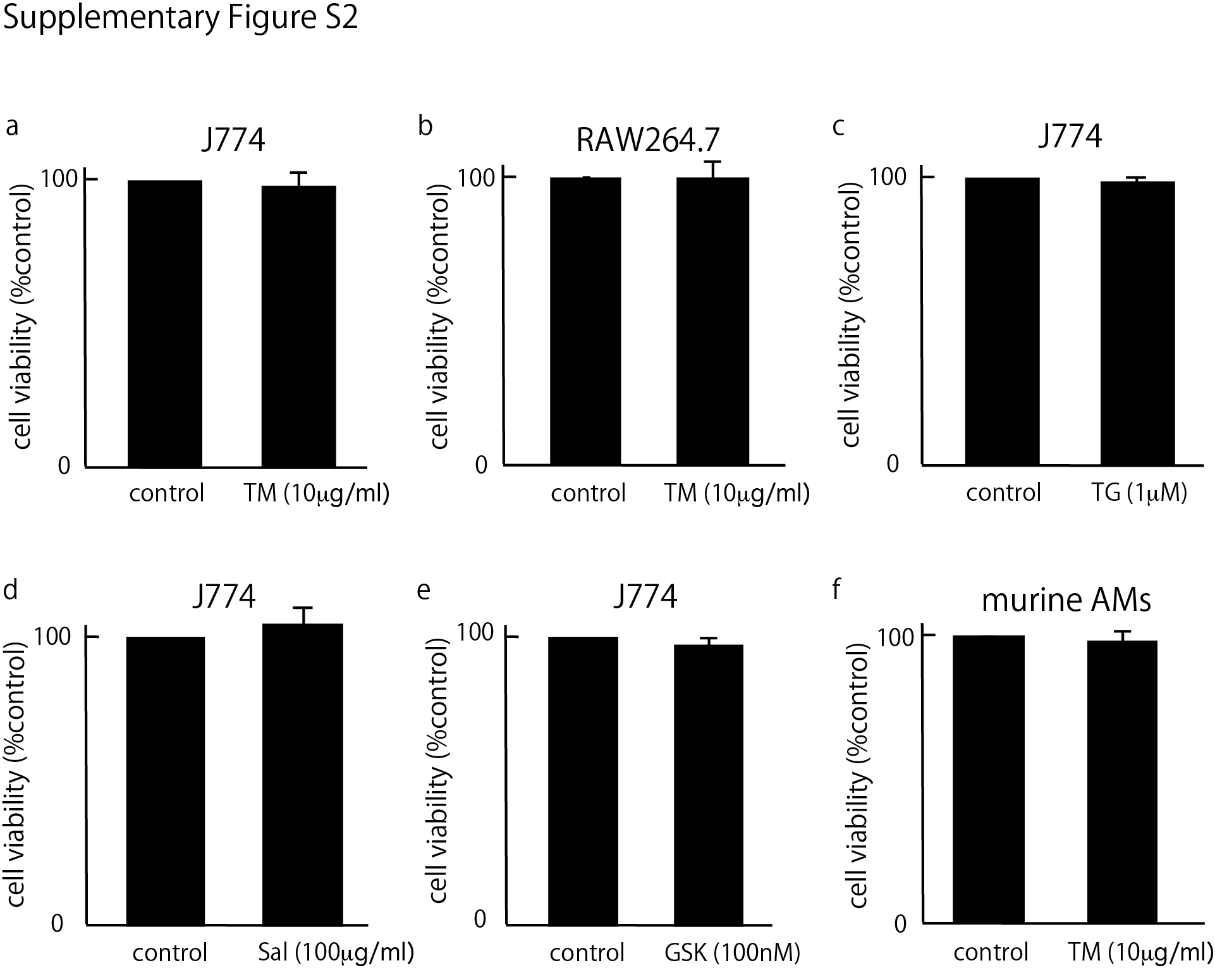


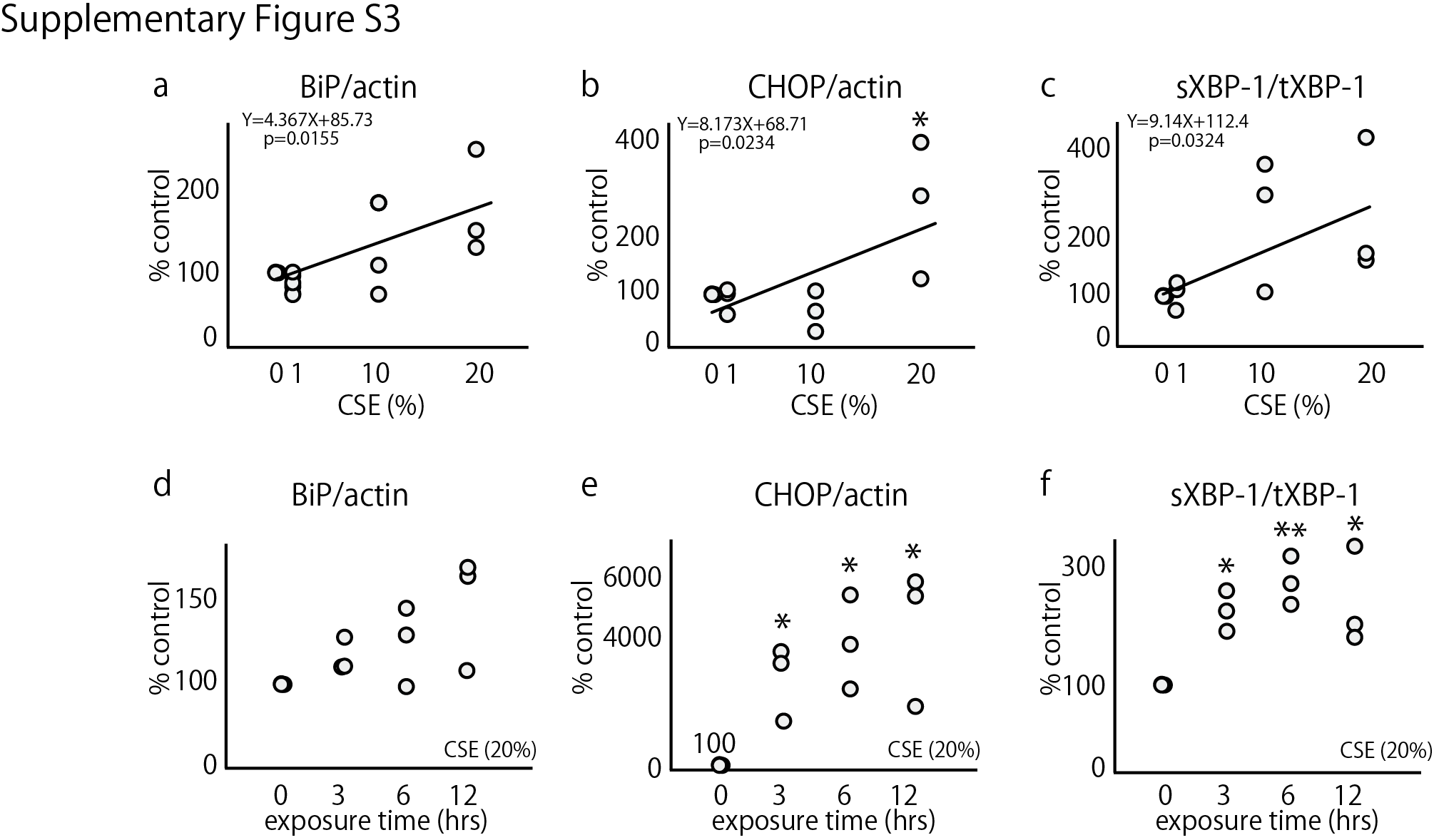


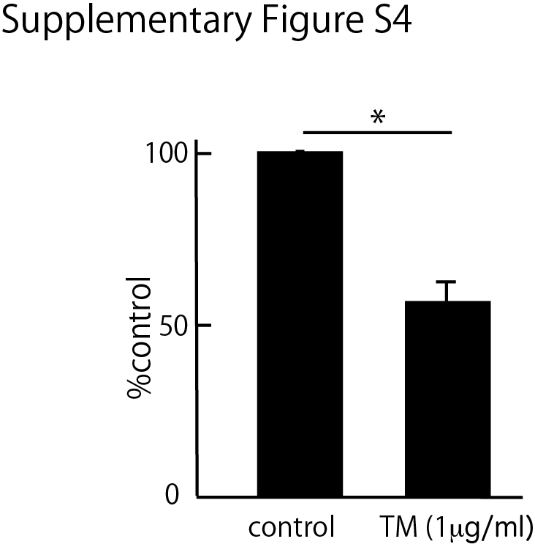

Supplement: Supplementary file 1 — Supplementary information [file 41598_2020_69610_MOESM1_ESM.docx]
